# Sero-neutralizing antibody against orthopoxviruses in populations of Wuhan, China

**DOI:** 10.1186/s43556-025-00319-x

**Published:** 2025-09-26

**Authors:** Zhiqiang Gao, Tianyu Liu, Zhenghao Zhao, Busen Wang, Lihua Hou

**Affiliations:** Laboratory of Advanced Biotechnology, Beijing Institute of Biotechnology, Beijing, People’s Republic of China

Dear editor,

In July 2022, the World Health Organization (WHO) classified monkeypox as a Public Health Emergency of International Concern. In the context of the global smallpox prevention campaign, vaccinia virus Tiantan strain (VVT) was utilized to vaccinate millions of individuals in China. Following the WHO 's declaration of smallpox eradication in 1979, China began phasing out nationwide smallpox vaccination in 1981. The eradication of smallpox and the subsequent discontinuation of vaccination efforts have led to a gradual decline in public immunity to orthopoxviruses, potentially increasing susceptibility to monkeypox virus infection. Consequently, it has become imperative to assess the residual orthopoxvirus-specific immunity within the Chinese population.

In this study, 450 serum samples were collected from individuals aged 18 to 80 in Wuhan, with 249 males and 201 females, yielding a male-to-female ratio of 1.24:1. Participants were grouped by birth decade, and their distribution and gender ratios are shown in Fig. 1c. This timeframe encompasses the period following the discontinuation of routine smallpox vaccination in China. The study’s serum sample collection and analysis procedures were ethically approved by our institution, with each subject voluntarily providing informed consent prior to participation.

**Fig. 1:**
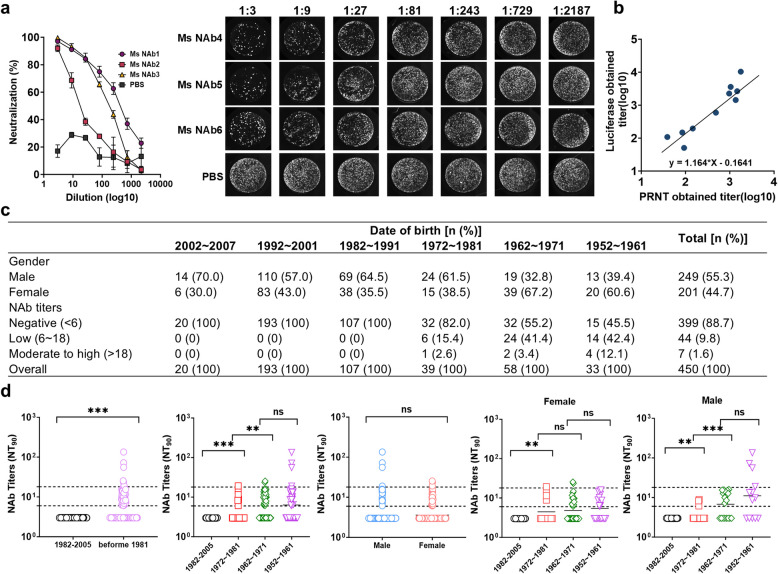
Seropositivity of NAb against Orthopoxvirus (*n* = 450). **a** Sera were collected from three mice vaccinated with MVA and PBS were tested using the MVA-Luc-eGFP neutralization assay. The x-axis indicates ranging from 1:3 to 1:2187. And the mouse serum was found to have a gradient inhibitory effect on eGFP expression. **b** The study compared neutralizing antibody titers (NT_90_) measured by two different neutralization assays, a luciferase-based inhibition assay (y-axis) and the plaque reduction neutralization test (PRNT, x-axis), with subsequent Spearman correlation analysis of the paired results from MVA-positive samples. **c** Demographic characteristics of serum sample donors and NAb positivity against orthopoxviruses. **d** NAb titers (NT_90_) were analyzed in vaccinated individuals born before 1981 (*n* = 137) and unvaccinated controls born after 1982 (*n* = 373), across six birth cohorts and by sex. *p < 0:05; **p < 0:01; ***p < 0.001, ns., not significant

We engineered a recombinant Modified Vaccinia Ankara (MVA) strain that encodes luciferase and enhanced green fluorescent protein (eGFP), designated as MVA-Luc-eGFP, and developed a luciferase-based virus neutralization assay using Vero-E6 cells. Neutralizing antibody titers (NAb) were determined by identifying the serum dilution that caused a 90% reduction in luciferase activity relative to negative controls (NT_90_), employing four-parameter nonlinear regression analysis in GraphPad software. NAb titers were categorized as follows: < 6 (negative), 6–18 (low), and > 18 (moderate to high). NAbs were detected in serum collected from the MVA-immunized mice, and NAbs in mouse serum were found to have a gradient inhibitory effect on luciferase and eGFP expression (Fig. 1a). To further validate the accuracy of this assay, we concurrently assessed 10 positive serum samples from MVA-immunized subjects using the luciferase expression inhibition assay and the plaque reduction neutralization test (PRNT). As anticipated, a strong correlation was observed between the serum titers (Spearman’s correlation test, *p*-value < 0.0001, Spearman *r* = 0.8745) (Fig. 1b). These findings indicate that the sero-neutralizing antibody against orthopoxviruses assay based on the inhibition of luciferase expression is stable and accurate.

Utilizing the cessation of smallpox vaccination in 1981 as a temporal demarcation, all individuals born after 1981 tested negative for serum anti-orthopoxvirus Nabs, demonstrating the test’s high specificity. As shown in Fig. 1c, 39.2% (51/130) of those born before 1981 were seropositive for NAbs, a significantly higher rate than those born after 1981 (Fig. 1d). Individuals born in 1972–1981 had the lowest seropositivity rate of 18.0% (7/39), with a 2.6% (1/39) proportion of moderate to high titer level antibodies; whereas those born in 1962–1971 had a seropositivity rate of 45.8% (26/58), with a 3.4%(2/58) proportion of moderate to high level antibodies; and those born in 1952–1961 had the the highest rate at 54.5%(18/33), and the proportion of moderate to high levels of NAbs was also highest at 12.1%(4/33). And the cohort 1962–1971 displayed significantly higher antibody levels and positivity rates compared to the 1972–1981 cohort, the 1952–1961 cohort had higher antibody levels than the 1962–1971 cohort (Fig. 1d). The study found no significant difference in serum NAb positivity between men and women in Wuhan. Among males born before 1981, NAb positivity increased with age whereas no significant difference was observed in NAb levels across age groups in females (Fig. 1d). Humoral immunity is crucial for protection, and individuals born before 1981 showed prolonged NAb levels lasting over 70 years.

Our data suggested that the positivity and the levels of anti-orthopoxvirus NAb were generally elevated in older age groups. In 2024, Min Li et al. [1] found that anti-VTT antibodies was significantly higher in individuals aged 70 years and older compared to those aged 41 to 70 years, however, that conclusion was Limited by a relatively small sample size of 60 participants in Beijing, China. The current study has expanded the sample to include 130 vaccinated individuals in another region, providing a more representative population. It has been reported that older individuals who received multiple doses of the smallpox vaccine showed higher antibody titers and a greater positive proportion against orthopoxviruses than subjects vaccinated once [2]. Another important reason may be the changes in strains and methods of vaccine production throughout China’s history, Vaccine production shifted from cattle (1949–1969) to chicken embryos post-1969. Between 1954 and 1964, the strain from Soviet replaced the Chinese Tiantan strain. Further research and investigation are required to ascertain whether the replacement of the vaccine strain and modifications in the production process have impacted vaccine efficacy. In addition, neutralising antibodies are considered to be the main indicator of protective immunity induced by smallpox vaccines. According to research by Mucker [3] and colleagues, antibody production triggered by smallpox vaccination proved essential and adequate to prevent fatal monkeypox infections in cynomolgus macaques. Several studies have demonstrated that threshold proposed based on data from the smallpox epidemic period serum (IC_50_ > 20 or > 32) are correlated with protective immunity against smallpox [4, 5]. This study indicates that individuals born after 1981, who have not received smallpox vaccination, exhibit an absence of neutralizing antibodies against orthopoxviruses and are therefore susceptible to monkeypox. It is recommended that prevention and control strategies prioritise young adults and high-risk behaviour groups. The NAb positivity rate of 54.5% and the high-titer proportion of 12.1% observed in the elderly cohort (1952–1961) were significantly elevated compared to younger cohorts. This suggests that repeated vaccinations may contribute to enhanced long-term immunity.

We acknowledge that our existing sampling methodology may not adequately reflect the socioeconomic and geographic diversity of Wuhan’s population. In subsequent research, we intend to employ a stratified sampling strategy to achieve more representative coverage across various regions (urban, suburban, and rural) and occupational/socioeconomic groups, thereby improving the generalizability of the results. It remains imperative to further elucidate the relationship between vaccine strains, the number of doses administered, and antibody persistence to inform immunization strategies. This study contributes significant immunological insights pertinent to monkeypox control, identifying a correlation between age and sero-neutralizing antibody against orthopoxviruses. The findings underscore the importance of long-term immunological surveillance of the orthopoxviruses within the Chinese population. Looking forward, it is crucial to enhance immunization coverage among high-risk groups and systematically evaluate the immune protection efficacy in historically vaccinated populations. This approach will facilitate the establishment of a dynamic control system to effectively address the public health challenges posed by the virus’s ongoing evolution.

## Supplementary Information

Supplementary Material 1.

## Data Availability

All data presented in this letter are included in the main text and the supplementary material, further inquiries can be directed to the corresponding authors.
