# Supplementary material for: Sero-neutralizing antibody against orthopoxviruses in populations of Wuhan, China
Source: Mol Biomed. 2025 Sep 26;6:68. doi: 10.1186/s43556-025-00319-x (PMC12463794; doi:10.1186/s43556-025-00319-x)
Supplement: Supplementary file 1 — Supplementary Material 1. [file 43556_2025_319_MOESM1_ESM.docx]

Supplementary Materials for

Sero-neutralizing antibody against orthopoxviruses in populations of Wuhan, China

Zhiqiang Gao^a,#^, Tianyu Liu^a^, Zhenghao Zhao^a^, Busen Wang^a,#,*^, Lihua Hou ^a,*^

^a^Laboratory of Advanced Biotechnology, Beijing Institute of Biotechnology, Beijing, People’s Republic of China

^#^These authors contributed equally

^*^Correspondence: [sen154034@163.com (B.W.)](mailto:sen154034@163.com (B.W.)), houlihua@sina.com(L.H.)

**Materials**

**Study design**

Serum samples were collected from 450 healthy adults living in Wuhan China. The participants' ages spanned from 18 to 80 years, covering the period subsequent to the cessation of routine smallpox vaccination in China. The collection and use of the serum samples for this study was approved by our institutional review boards, and informed consent was obtained from each participant.

**Mouse serum, virus and cells**

Samples from MVA virus-vaccinated mice were kindly provided by SINO ANIMAL (Beijing, China). The recombinant poxvirus MVA-Luc-eGFP, generated using the established MVA reverse genetics system^1^, which stably expresses luciferase and enhanced green fluorescent protein, was used to detect sero-neutralizing antibody titer on the Vero-E6 cell line. And the PRNT method was performed on the BHK-21 cell line.

**Methods**

**Luciferase-based neutralization assay**

Sero-neutralizing antibody against orthopoxviruses antibodies were detected using the recombinant poxvirus MVA-Luc-eGFP. In the serum neutralization assay, samples were heat-inactivated at 56℃ for 60 min. The final serum dilutions were 1:6, 1:18, 1:54, 1:162, and 1:486, and serum-free dilution was used as a negative control. Subsequently, 50 μL of each serum dilution was mixed with 50 μL of MVA-Luc-eGFP and incubated at 37°C for 1 h. Next, 100 μL of 2×10^5^ Vero-E6 cells were added to the mixture and incubated at 37°C for 24 h prior to the measurement of luciferase activity using the Bright-Lite luciferase assay system (Cat No. DD1204-03, Vazyme, Nanjing, China). The neutralizing titer, indicative of 90% neutralization potency, was determined using the Reed–Muench method as the reciprocal of the dilution at which luciferase activity was reduced to 10% of that observed in the negative control, and the NAb titer of negative serum was defined as 3, which is half of the minimum dilution.

**Plaque reduction neutralization test (PRNT)**

Sera were subjected to a series of five three-fold dilutions, ranging from 1:6 to 1:486, based on the anticipated titer. Each dilution, in a volume of 550 µL, was combined with an equal volume of MVA-Luc-eGFP virus (80-100 IFU/0.5 mL.). Following incubation at 37°C with 5% CO_2_ for 1 h, 500 µL of the mixture was added to BHK-21 cells in duplicate wells. The plates were then incubated for 2 h at 37°C with 5% CO_2_, with intermittent rocking, before the addition of 3 mL of overlay medium containing 0.5% agarose. On the fourth day, viral plaques were visualized by staining with 0.1% crystal violet in PBS containing 0.2% formaldehyde, and subsequently counted. The neutralization titer 90 (NT_90_) was determined as the reciprocal of the serum dilution required to achieve a 90% reduction in MVA-Luc-eGFP plaques, and the NAb titer of negative serum was also defined as 3.

**Data analysis**

The analysis was performed with GraphPad Prism v.7.0. Unpaired t-tests were conducted to compare differences between two experimental groups. One-way ANOVA with Tukey’s multiple comparisons tests were applied to compare more than two experimental groups. A p-value of less than 0.05 was considered statistically significant, with significance levels denoted as follows: **p*<0.05; ***p*<0.01; ****p*<0.001; and ns. for not significant. The antibody titer data were log transformed before analysis. The error bars throughout all the figures represent one standard deviation.

**References**

1. Gao, Z.; Wang, B.; Liu, T.; Zhao, Z.; Xu, J.; Zhao, X., et al., Design and construction of a fast synthetic modified vaccinia virus Ankara reverse genetics system for advancing vaccine development. Frontiers in microbiology **2025,** 16, 1572706. DOI:10.3389/fmicb.2025.1572706.
